# Supplementary material for: Downregulation of mitochondrial biogenesis by virus infection triggers antiviral responses by cyclic GMP-AMP synthase
Source: PLoS Pathog. 2021 Oct 14;17(10):e1009841. doi: 10.1371/journal.ppat.1009841 (PMC8516216; doi:10.1371/journal.ppat.1009841)
Supplement: S1 Table — (PDF) [file ppat.1009841.s007.pdf]

S1 Table: List of antibidies, siRNAs and primer pairs used in this study

Antibodies

antibody	maker	cat. No
anti-COX IV mouse monoclonal antibody	abcam	ab33985
anti-VSV NewJersey Glycoprotein rabbit antiserum	Alpha Diagnostic International	VSNG13-S
anti-Sendai virus rabbit antiserum	MBL	PD029
anti-RSV nucleoprotein mouse monoclonal antibody	abcam	ab94806
anti-vaccinia virus rabbit polyclonal antibody	abcam	ab35219
anti-HA-tag mouse monoclonal antibody	SIGMA-ALDRICH	H90658
anti-HSP70 mouse monoclonal antibody	Enzo	ADI-SPA-810-D
anti-GAPDH mouse monoclonal antibody	Millipore	MAB374
anti-PDI rabbit polyclonal antibody	Stressgen	SPA-890
anti-histone H3 rabbit polyclonal antibody	Biovision	3624-100
anti-PGC1alpha rabbit polyclonal antibody	abcam	ab54481
anti-MAVS rabbit polyclonal antibody	abcam	ab31334
anti-STING mouse monoclonal antibody	Proteintech	66680-1-Ig
anti-p-STING (S366) rabbit monoclonal antibody	Cell signaling technology	D7C3S
mouse IgG Isotype	R&D	11711
Alexa488 donkey anti-mouse IgG	Invitrogen	A21202
Alexa488 goat anti-rabbit IgG	Invitrogen	A11008
Alexa568 goat anti-mouse IgG	Invitrogen	A11019
Alexa405 goat anti-rabbit IgG	Abcam	ab175651

siRNA

	Official symbol	Accession ID	sense	antisensed	Official full name	homology
human						
cGAS	CGAS	NM_138441	GCACGUGAAGAUUUCUGCACCUAAU	AUUAGGUGCAGAAAUCUUCACGUGC	cyclic GMP-AMP synthase	
MAVS	MAVS	NM_020746	CCGUUUGCUGAAGACAAGACCUAUA	UAUAGGUCUUGUCUUCAGCAAACGG	mitochondrial antiviral signaling protein	
PGC-1α	PPARGC1A	NM_001330751	GAACGACUUGGAUACAGACAGCUUU	AAAGCUGUCUGUAUCCAAGUCGUUC	PPARG coactivator 1 alpha	
Mfn1	MFN1	NM_033540	CAGAGCUGGAUAGCUGGAUUGAUAA	UUAUCAAUCCAGCUAUCCAGCUCUG	mitofusin 1	african green monkey

S1 Table (continued)

qPCR primer pairs

	Official symbol	ID	Forward primer	Reverse primer	Official full name	homolgy
<u>human</u>						
28S rRNA	RNA28SN1	NR_145822.1	CGACGTCGCTTTTTGATCC	GCAACAACACATCATCAGTAG	28S ribosomal N1, ribosomal RNA	mouse, african green monkey, dog
IFN-β	IFNB1	NM_002176	ATGACCAACAAGTGTCTCCTC	GCCACAGGAGCTTCTGACAC	interferon beta 1	
cxcl10	CXCL10	NM_001565.3	CCTGCAAGCCAATTTTGTCC	GATGGCCTTCGATTCTGGAT	C-X-C motif chemokine ligand 10	
Ifi44	IFI44	NM_006417.4	AGGTCTGTTTTCCAAGGGCA	TTGCCATCTTTCCCGTCTCT	interferon induced protein 44	
Ifit2	IFIT2	NM_001547.4	TGAAAGAGCGAAGGTGTGCT	AGAGGGTCAATGGGTTCTG	interferon induced protein with tetratricopeptide repeats 2	
Mx2	MX2	NM_002463.1	TGAACGTGCAGCGAGCTT	TGGTGTTCACCATCTCGTCG	MX dynamin like GTPase 2	
OAS1	OAS1	NM_016816.3	GGTGGAGTTCGATGTGCTG	AGGTTTATAGCCGCCAGTCA	2'-5'-oligoadenylate synthetase 1	
OAS2	OAS2	NM_016817.2	CCAGCTGAGAGCAATGGGAA	TGGTGTTCACCATCTCGTCG	2'-5'-oligoadenylate synthetase 2	
STAT1	STAT1	NM_007315.3	CATTCCGTGGACGAGGTTTT	GATGCACCCATCATTCCAGA	signal transducer and activator of transcription 1	
IFN-α	IFNA1	NM_024013.2	GATGCTCCTGGCACAAATGA	CTGGAACTGGTTGCCATCAA	interferon alpha 1	
H2AFL	HIST1H2AC	NM_003512.3	ATGTCTGGACGTGGTAAGC	GTAGTTGCCTTTACGGAGC	histone cluster 1 H2A family member c	
JAK1	JAK1	NM_002227.3	AGAGGACTGCAATGCCATGG	CCGACAGATAGAAGATCAC	Janus kinase 1	
LAMA4	LAMA4	NM_001105206.2	ATGGCTTTGAGCTCAGCCTG	TGAGCTCCCTTCAATGTC	laminin subunit alpha 4	
ATP1A1	ATP1A1	NM_000701.7	AATTCGGTCTTCCAGCAGGG	GTAGGGGAAGGCACAGAACC	ATPase Na+/K+ transporting subunit alpha 1	
SLC27A4	SLC27A4	NM_005094.3	GTCCTCCTGAAGGTGAAGGC	CCTGCAGGAAGTTGGCTAC	solute carrier family 27 member 4	
MAPK6	MAPK6	NM_002748	ATTCATGGTTTTGATCTGGG	GCTACTCTTTTGTCACAGTC	mitogen-activated protein kinase 6	
ATP5G3	ATP5G3	NM_001689	ATGTTCGCCTGCGCCAAG	CTCTCCAGTCCTACTAGC	ATP synthase, H+ transporting, mitochondrial Fo complex, subunit C3	
MDH1	MDH1	NM_005917.3	TCCTGGACGGTGTCCTAATGG	GCAACGTCTTCTTTATCTGTTGCG	malate dehydrogenase 1	
MRPL43	MRPL43	NM_032112.2	GACTTCGCCCGACGGAATC	GTATTCGGCCACTACTCTGGG	mitochondrial ribosomal protein L43	
NDUFA8	NDUFA8	NM_014222.2	CCCAACAAGGAGTTTATGCTCT	CACAGTGACGTTTTATCTGCCT	NADH:ubiquinone oxidoreductase subunit A8	
NDUFB4	NDUFB4	NM_004547	ATGTCGTTCCCAAAGTATAAGCCG	TCTTATGGCCAACCGCTC	NADH dehydrogenase (ubiquinone) 1 beta subcomplex, 4	
ITGAM	ITGAM	NM_000632.3	AGGACTCTGCCCAGACCAC	GTCGGGGATACTTCGCTGT	integrin subunit alpha M	
PGC-1alpha	PPARGC1A	NM_001330751.2	TGAAGGGTACTTTTCTGCCCC	GCACAAACTGGATTCGCCAG	PPARG coactivator 1 alpha	
TFAM	TFAM	NM_003201.3	CCAAAAAGACCTCGTTCAGCTT	CTTCAGCTTTTCCTGCGGTG	transcription factor A, mitochondrial	
mtDNA D-loop		MH980012	GACCACCATCCTCCGTGAAATC	TCACTTTAGCTACCCCCAAGTGTTA	(amplicon location : 16397-16485 in 16568)	
mtDNA 1			CTATCACCCTATTAACCACTCA	TTCGCCTGTAATATTGAACGTA	(11-189)	
mtDNA 2			CACCCAAGAACAGGGTTTGT	TGGCCATGGGTATGTTGTTAA	(3211-3319)	
mtDNA 3			AATCGAGTAGTACTCCCCGATTG	TTCTAGGACGATGGGCATGAAA	(8002-8220)	
mtDNA 4			AATCCAAGCCTACGTTTTCACA	AGTATGAGGAGCGTTATGGAGT	(9149-9339)	
<u>mouse</u>						
mIFN-b	Ifnb1	NM_010510.1	CTGGAGCAGCTGAATGGAAA	ATCTCTTGGATGGCAAAGGC	interferon beta 1	
mIfit1	Ifit1	NM_008331.3	CAGCAGCACATCTTGCCAAA	GAGGAAGGTGATGCCTGCAA	interferon-induced protein with tetratricopeptide repeats 1	
mIfit2	Ifit2	NM_008332.3	AGATGAGACTTAGAGGTGCTGC	TGTGTCAAAGCGCTCAAAGC	interferon-induced protein with tetratricopeptide repeats 2	
mIfit3	Ifit3	NM_010501.2	TGCTTTGGGGAAAGCTCCTA	TGTGGATCGCAGTGCTTTTC	interferon-induced protein with tetratricopeptide repeats 3	
mIFN-a1	Ifna1	NM_010502.2	GATGCCCAGCAGATCAAGAA	TGGTGGAGGTCATTGCAGAA	interferon alpha 1	
mISG15	Isg15	NM_015783.3	TAAAGGTGAAGATGCTGGGG	CACACCAATCTTCTGGGCAA	ISG15 ubiquitin-like modifier	
mMx2	Mx2	NM_013606	GTCGCCTATTCACCAGGCTC	AACCCTGGCAATTCTCGTCC	MX dynamin-like GTPase 2	
mOAS2	Oas2	NM_145227.3	GCTGGTGCTGGCATCTTCTA	GGGGTCTGCATTACTGGCAC	2'-5' oligoadenylate synthetase 2	
mSTAT1	Stat1	NM_009283.4	ATGGAAGAATCCACCAACGG	CTGGCACAACTGGGTTTCAA	signal transducer and activator of transcription 1	
