# Supplementary material for: Downregulation of mitochondrial biogenesis by virus infection triggers antiviral responses by cyclic GMP-AMP synthase
Source: PLoS Pathog. 2021 Oct 14;17(10):e1009841. doi: 10.1371/journal.ppat.1009841 (PMC8516216; doi:10.1371/journal.ppat.1009841)
Supplement: S2 Table — (PDF) [file ppat.1009841.s008.pdf]

S2 Table: Entire lists of the results of gene ontology analysis

MeV

GO cellular component complete	reference genes #	#	expected	Fold Enrichment	+/-	P value
endoplasmic reticulum chaperone complex	11	5	0.23	22.09	+	5.67E-03
↳endoplasmic reticulum part	1340	70	27.57	2.54	+	1.42E-09
↳cytoplasmic part	9646	324	198.49	1.63	+	1.82E-31
↳intracellular part	14422	397	296.77	1.34	+	5.31E-28
↳intracellular	14699	398	302.47	1.32	+	4.59E-26
↳cell part	17007	416	349.97	1.19	+	5.08E-18
↳cell	17034	416	350.52	1.19	+	8.77E-18
↳cytoplasm	11495	360	236.54	1.52	+	5.06E-33
↳intracellular organelle part	9183	315	188.97	1.67	+	1.83E-31
↳intracellular organelle	12625	363	259.8	1.4	+	6.61E-24
↳organelle	13455	385	276.88	1.39	+	3.21E-29
↳organelle part	9480	316	195.08	1.62	+	6.46E-29
↳endoplasmic reticulum	1888	89	38.85	2.29	+	2.09E-10
↳intracellular membrane-bounded organelle	10846	331	223.19	1.48	+	1.87E-23
↳membrane-bounded organelle	12364	374	254.43	1.47	+	6.44E-33
↳endomembrane system	4432	168	91.2	1.84	+	4.95E-14
↳protein-containing complex	5383	200	110.77	1.81	+	2.76E-17
mitochondrial proton-transporting ATP synthase complex	23	6	0.47	12.68	+	1.43E-02
↳inner mitochondrial membrane protein complex	147	16	3.02	5.29	+	1.62E-04
↳mitochondrial inner membrane	443	30	9.12	3.29	+	3.09E-05
↳mitochondrial membrane	674	40	13.87	2.88	+	5.25E-06
↳organelle membrane	2935	114	60.4	1.89	+	1.29E-08
↳membrane	9711	269	199.83	1.35	+	2.51E-08
↳mitochondrial envelope	713	42	14.67	2.86	+	2.59E-06
↳mitochondrial part	1008	58	20.74	2.8	+	3.90E-09
↳mitochondrion	1568	81	32.27	2.51	+	2.93E-11
↳organelle envelope	1161	57	23.89	2.39	+	2.30E-06
↳envelope	1161	57	23.89	2.39	+	2.30E-06
↳organelle inner membrane	503	32	10.35	3.09	+	4.33E-05
↳mitochondrial membrane part	235	19	4.84	3.93	+	9.77E-04
↳mitochondrial protein complex	279	21	5.74	3.66	+	7.98E-04
↳proton-transporting ATP synthase complex	24	6	0.49	12.15	+	1.81E-02
↳proton-transporting two-sector ATPase complex	53	8	1.09	7.34	+	2.55E-02
melanosome	105	24	2.16	11.11	+	1.79E-14
↳pigment granule	105	24	2.16	11.11	+	1.79E-14
↳cytoplasmic vesicle	2289	112	47.1	2.38	+	3.25E-15
↳intracellular vesicle	2292	112	47.16	2.37	+	3.58E-15
↳vesicle	3783	195	77.85	2.5	+	3.00E-35
cytosolic large ribosomal subunit	66	14	1.36	10.31	+	2.80E-07
↳cytosolic part	259	33	5.33	6.19	+	3.68E-13
↳cytosol	5077	172	104.47	1.65	+	7.30E-10
↳large ribosomal subunit	125	17	2.57	6.61	+	2.65E-06
↳ribosomal subunit	199	26	4.1	6.35	+	3.29E-10
↳ribonucleoprotein complex	871	57	17.92	3.18	+	3.55E-11
↳ribosome	252	27	5.19	5.21	+	1.03E-08
↳intracellular non-membrane-bounded organelle	4197	134	86.37	1.55	+	5.53E-05
↳non-membrane-bounded organelle	4204	134	86.51	1.55	+	6.12E-05
↳cytosolic ribosome	123	23	2.53	9.09	+	5.71E-12
COP9 signalosome	37	7	0.76	9.19	+	2.06E-02
↳nuclear part	4488	146	92.35	1.58	+	2.51E-06
↳nucleus	7295	203	150.12	1.35	+	1.54E-04
cytosolic small ribosomal subunit	49	9	1.01	8.93	+	1.59E-03
myelin sheath	163	28	3.35	8.35	+	4.75E-14
ficolin-1-rich granule lumen	124	21	2.55	8.23	+	5.81E-10
↳ficolin-1-rich granule	185	27	3.81	7.09	+	8.54E-12
↳secretory granule	835	63	17.18	3.67	+	1.64E-15
↳secretory vesicle	986	69	20.29	3.4	+	1.48E-15
↳cytoplasmic vesicle part	1475	70	30.35	2.31	+	1.03E-07
↳intracellular organelle lumen	5222	198	107.46	1.84	+	4.52E-18
↳organelle lumen	5222	198	107.46	1.84	+	4.52E-18
↳membrane-enclosed lumen	5222	198	107.46	1.84	+	4.52E-18
polysome	76	12	1.56	7.67	+	1.33E-04
azurophil granule membrane	58	9	1.19	7.54	+	6.17E-03
↳lysosomal membrane	345	22	7.1	3.1	+	6.16E-03

↳lysosome	669	44	13.77	3.2	+	3.44E-08
↳lytic vacuole	670	44	13.79	3.19	+	3.60E-08
↳vacuole	771	47	15.87	2.96	+	8.56E-08
↳lytic vacuole membrane	346	22	7.12	3.09	+	6.45E-03
↳vacuolar membrane	402	23	8.27	2.78	+	2.10E-02
↳whole membrane	1635	66	33.64	1.96	+	1.96E-04
↳vacuolar part	557	37	11.46	3.23	+	1.11E-06
↳bounding membrane of organelle	2068	74	42.56	1.74	+	3.02E-03
↳azurophil granule	154	21	3.17	6.63	+	3.14E-08
↳primary lysosome	154	21	3.17	6.63	+	3.14E-08
↳secretory granule membrane	300	22	6.17	3.56	+	6.60E-04
rough endoplasmic reticulum	92	12	1.89	6.34	+	9.85E-04
azurophil granule lumen	90	11	1.85	5.94	+	5.27E-03
↳vacuolar lumen	170	17	3.5	4.86	+	2.14E-04
↳secretory granule lumen	320	30	6.58	4.56	+	1.78E-08
↳cytoplasmic vesicle lumen	336	30	6.91	4.34	+	5.71E-08
↳vesicle lumen	337	30	6.93	4.33	+	6.12E-08
mitochondrial respiratory chain	95	11	1.95	5.63	+	8.72E-03
↳respiratory chain	105	11	2.16	5.09	+	2.19E-02
oxidoreductase complex	113	13	2.33	5.59	+	1.36E-03
↳catalytic complex	1413	68	29.08	2.34	+	1.12E-07
focal adhesion	404	45	8.31	5.41	+	1.76E-16
↳cell-substrate adherens junction	407	45	8.38	5.37	+	2.32E-16
↳adherens junction	538	50	11.07	4.52	+	2.42E-15
↳anchoring junction	554	52	11.4	4.56	+	3.13E-16
↳cell junction	1276	65	26.26	2.48	+	3.02E-08
↳cell-substrate junction	411	45	8.46	5.32	+	3.35E-16
respiratory chain complex	90	10	1.85	5.4	+	3.24E-02
lysosomal lumen	94	10	1.93	5.17	+	4.66E-02
endoplasmic reticulum-Golgi intermediate compartment	126	13	2.59	5.01	+	4.42E-03
endoplasmic reticulum lumen	300	29	6.17	4.7	+	1.93E-08
extracellular exosome	2095	154	43.11	3.57	+	1.76E-43
↳extracellular vesicle	2115	154	43.52	3.54	+	5.67E-43
↳extracellular organelle	2117	154	43.56	3.54	+	6.37E-43
↳extracellular region part	3491	179	71.84	2.49	+	5.46E-31
↳extracellular region	4302	190	88.53	2.15	+	5.18E-25
↳extracellular space	3304	173	67.99	2.54	+	1.05E-30
cell-cell junction	452	26	9.3	2.8	+	5.32E-03
mitochondrial matrix	466	26	9.59	2.71	+	9.06E-03
endoplasmic reticulum membrane	1062	47	21.85	2.15	+	1.33E-03
↳nuclear outer membrane-endoplasmic reticulum membrane network	1085	48	22.33	2.15	+	1.02E-03
↳endoplasmic reticulum subcompartment	1067	47	21.96	2.14	+	1.51E-03
↳organelle subcompartment	1687	60	34.71	1.73	+	3.82E-02
synapse	1176	50	24.2	2.07	+	1.83E-03
nucleoplasm	3486	106	71.73	1.48	+	2.34E-02
↳nuclear lumen	4090	130	84.16	1.54	+	1.29E-04
Unclassified	2312	6	47.58	0.13	-	0.00E+00

RSV

GO cellular component complete	reference genes #	#	expected	Fold Enrichment	+/-	P value
ciliary tip	46	39	18.94	2.06	+	4.95E-02
↳ciliary part	445	284	183.25	1.55	+	2.64E-09
↳cilium	595	377	245.02	1.54	+	1.68E-12
↳organelle	13455	6486	5540.71	1.17	+	6.61E-102
↳plasma membrane bounded cell projection	2078	1000	855.71	1.17	+	2.93E-04
↳cell projection	2145	1033	883.3	1.17	+	1.58E-04
↳cell part	17007	7520	7003.41	1.07	+	2.71E-46
↳cell	17034	7528	7014.52	1.07	+	5.63E-46
↳organelle part	9480	4702	3903.82	1.2	+	3.30E-63
↳plasma membrane bounded cell projection part	1443	711	594.22	1.2	+	1.06E-03
↳cell projection part	1443	711	594.22	1.2	+	1.06E-03
ciliary transition zone	69	57	28.41	2.01	+	2.08E-03
centriole	134	103	55.18	1.87	+	7.23E-06
↳microtubule organizing center part	181	136	74.53	1.82	+	1.25E-07
↳microtubule organizing center	732	474	301.43	1.57	+	6.74E-18
↳microtubule cytoskeleton	1191	729	490.45	1.49	+	2.42E-22
↳cytoskeleton	2168	1104	892.77	1.24	+	3.80E-10
↳intracellular non-membrane-bounded organelle	4197	2089	1728.31	1.21	+	2.25E-18
↳non-membrane-bounded organelle	4204	2094	1731.19	1.21	+	1.41E-18
↳intracellular organelle	12625	6137	5198.92	1.18	+	8.03E-95
↳intracellular part	14422	6954	5938.91	1.17	+	1.23E-129
↳intracellular	14699	7024	6052.98	1.16	+	8.61E-122
↳cytoskeletal part	1673	880	688.93	1.28	+	2.24E-10
↳intracellular organelle part	9183	4588	3781.52	1.21	+	9.64E-65
↳cytoplasmic part	9646	4746	3972.18	1.19	+	2.76E-59
↳cytoplasm	11495	5578	4733.59	1.18	+	1.27E-72
ciliary basal body	119	91	49	1.86	+	6.73E-05
axoneme	114	85	46.94	1.81	+	4.97E-04
↳ciliary plasm	115	86	47.36	1.82	+	3.79E-04
↳plasma membrane bounded cell projection cytoplasm	194	135	79.89	1.69	+	1.45E-05
↳cytoplasmic region	492	263	202.6	1.3	+	3.10E-02
peroxisomal part	101	73	41.59	1.76	+	8.89E-03
↳peroxisome	135	95	55.59	1.71	+	1.25E-03
↳microbody	135	95	55.59	1.71	+	1.25E-03
↳intracellular membrane-bounded organelle	10846	5374	4466.33	1.2	+	8.02E-83
↳membrane-bounded organelle	12364	5979	5091.44	1.17	+	2.23E-83
↳microbody part	101	73	41.59	1.76	+	8.89E-03
spindle pole	152	104	62.59	1.66	+	1.36E-03
↳spindle	335	217	137.95	1.57	+	3.09E-07
cullin-RING ubiquitin ligase complex	158	107	65.06	1.64	+	1.51E-03
↳ubiquitin ligase complex	283	172	116.54	1.48	+	1.09E-03
↳transferase complex	813	470	334.79	1.4	+	8.76E-10
↳catalytic complex	1413	761	581.87	1.31	+	1.40E-10
↳protein-containing complex	5383	2491	2216.69	1.12	+	1.96E-08
kinetochore	134	89	55.18	1.61	+	2.29E-02
↳chromosomal part	884	502	364.03	1.38	+	2.01E-09
↳chromosome	1011	567	416.33	1.36	+	5.11E-10
↳chromosome, centromeric region	192	122	79.06	1.54	+	5.77E-03
↳chromosomal region	331	203	136.3	1.49	+	6.34E-05
centrosome	517	341	212.9	1.6	+	2.24E-13
mitochondrial matrix	466	306	191.9	1.59	+	1.37E-11
↳intracellular organelle lumen	5222	2643	2150.4	1.23	+	3.98E-30
↳organelle lumen	5222	2643	2150.4	1.23	+	3.98E-30
↳membrane-enclosed lumen	5222	2643	2150.4	1.23	+	3.98E-30
↳mitochondrial part	1008	591	415.09	1.42	+	5.89E-14
↳mitochondrion	1568	896	645.7	1.39	+	2.10E-19
motile cilium	180	115	74.12	1.55	+	8.44E-03
microtubule	422	268	173.78	1.54	+	1.78E-08
mitochondrial inner membrane	443	260	182.43	1.43	+	3.79E-05
↳mitochondrial membrane	674	375	277.55	1.35	+	1.26E-05
↳organelle membrane	2935	1486	1208.62	1.23	+	5.27E-14
↳mitochondrial envelope	713	394	293.61	1.34	+	1.10E-05
↳organelle envelope	1161	625	478.09	1.31	+	2.89E-08
↳envelope	1161	625	478.09	1.31	+	2.89E-08
↳organelle inner membrane	503	291	207.13	1.4	+	2.10E-05
transferase complex, transferring phosphorus-containing groups	283	163	116.54	1.4	+	3.46E-02

chromatin	512	285	210.84	1.35	+	7.10E-04
nuclear chromosome part	539	299	221.96	1.35	+	5.00E-04
↳nuclear part	4488	2311	1848.14	1.25	+	2.77E-29
↳nucleus	7295	3624	3004.05	1.21	+	2.04E-40
↳nuclear chromosome	577	320	237.61	1.35	+	2.01E-04
↳nuclear lumen	4090	2116	1684.24	1.26	+	4.09E-27
nuclear body	747	407	307.61	1.32	+	2.88E-05
↳nucleoplasm part	1109	626	456.68	1.37	+	8.70E-12
↳nucleoplasm	3486	1854	1435.52	1.29	+	1.36E-28
Golgi subcompartment	844	427	347.56	1.23	+	2.00E-02
↳Golgi apparatus part	963	480	396.56	1.21	+	2.44E-02
↳Golgi apparatus	1543	775	635.4	1.22	+	1.89E-05
↳endomembrane system	4432	2036	1825.08	1.12	+	3.14E-05
↳organelle subcompartment	1687	848	694.7	1.22	+	3.18E-06
cytosol	5077	2540	2090.69	1.21	+	2.08E-25
endoplasmic reticulum membrane	1062	521	437.33	1.19	+	4.97E-02
↳nuclear outer membrane-endoplasmic reticulum membrane network	1085	534	446.8	1.2	+	2.92E-02
↳endoplasmic reticulum subcompartment	1067	524	439.39	1.19	+	4.28E-02
bounding membrane of organelle	2068	1013	851.59	1.19	+	1.07E-05
integral component of plasma membrane	1618	516	666.29	0.77	-	2.65E-07
↳intrinsic component of membrane	5647	2159	2325.41	0.93	-	3.59E-02
↳intrinsic component of plasma membrane	1698	553	699.23	0.79	-	1.98E-06
↳plasma membrane part	2880	1030	1185.97	0.87	-	5.23E-04
↳plasma membrane	5575	1869	2295.76	0.81	-	2.04E-23
↳cell periphery	5695	1924	2345.18	0.82	-	2.11E-22
extracellular matrix	433	127	178.31	0.71	-	3.84E-02
↳extracellular region part	3491	1228	1437.58	0.85	-	5.13E-07
↳extracellular region	4302	1472	1771.54	0.83	-	2.69E-13
Unclassified	2312	636	952.07	0.67	-	0.00E+00
external side of plasma membrane	385	89	158.54	0.56	-	1.31E-06
↳side of membrane	584	175	240.49	0.73	-	6.06E-03
↳cell surface	880	261	362.38	0.72	-	9.58E-06
keratin filament	102	9	42	0.21	-	1.08E-06
↳intermediate filament	214	35	88.12	0.4	-	1.22E-07
↳intermediate filament cytoskeleton	251	48	103.36	0.46	-	1.10E-06
immunoglobulin complex, circulating	95	3	39.12	0.08	-	1.45E-10
↳extracellular space	3304	1152	1360.57	0.85	-	2.43E-07
↳immunoglobulin complex	100	4	41.18	0.1	-	2.25E-10

VSV

GO cellular component complete	reference genes #	#	expected	Fold Enrichment	+/-	P value
centrosome	517	32	12.01	2.66	+	1.19E-03
↳microtubule organizing center	732	36	17.01	2.12	+	3.91E-02
↳organelle	13455	371	312.68	1.19	+	1.42E-05
↳intracellular organelle	12625	368	293.4	1.25	+	1.23E-09
↳intracellular part	14422	406	335.16	1.21	+	3.28E-10
↳intracellular	14699	408	341.59	1.19	+	4.82E-09
↳intracellular organelle part	9183	278	213.41	1.3	+	4.32E-06
↳organelle part	9480	281	220.31	1.28	+	3.47E-05
nuclear body	747	45	17.36	2.59	+	1.45E-05
↳nucleoplasm part	1109	56	25.77	2.17	+	8.97E-05
↳nucleoplasm	3486	168	81.01	2.07	+	1.40E-18
↳nuclear lumen	4090	180	95.05	1.89	+	4.24E-16
↳intracellular organelle lumen	5222	197	121.36	1.62	+	5.82E-11
↳organelle lumen	5222	197	121.36	1.62	+	5.82E-11
↳membrane-enclosed lumen	5222	197	121.36	1.62	+	5.82E-11
↳nuclear part	4488	188	104.3	1.8	+	8.52E-15
↳nucleus	7295	269	169.53	1.59	+	4.56E-17
↳intracellular membrane-bounded organelle	10846	335	252.05	1.33	+	2.84E-11
↳membrane-bounded organelle	12364	348	287.33	1.21	+	1.19E-05
cytosol	5077	170	117.99	1.44	+	1.18E-04
Unclassified	2312	41	53.73	0.76	-	0.00E+00
extracellular space	3304	42	76.78	0.55	-	3.39E-03
↳extracellular region part	3491	46	81.13	0.57	-	4.88E-03
↳extracellular region	4302	54	99.98	0.54	-	3.39E-05
integral component of plasma membrane	1618	14	37.6	0.37	-	7.18E-03
↳integral component of membrane	5488	81	127.54	0.64	-	4.94E-04
↳intrinsic component of membrane	5647	81	131.23	0.62	-	7.22E-05
↳membrane part	6959	114	161.72	0.7	-	2.12E-03
↳membrane	9711	169	225.68	0.75	-	1.86E-04
↳intrinsic component of plasma membrane	1698	14	39.46	0.35	-	1.94E-03
↳plasma membrane part	2880	36	66.93	0.54	-	1.15E-02
↳plasma membrane	5575	82	129.56	0.63	-	3.19E-04
↳cell periphery	5695	83	132.35	0.63	-	1.37E-04

SeV

GO cellular component complete	reference genes #	#	expected	Fold Enrichment	+/-	P value
synaptic vesicle	184	9	1.57	5.75	+	4.71E-02
↳exocytic vesicle	202	10	1.72	5.82	+	1.55E-02
Unclassified	2312	10	19.67	0.51	-	0.00E+00

MVA

GO cellular component complete	reference genes #	#	expected	Fold Enrichment	+/-	P value
mitochondrial proton-transporting ATP synthase complex, coupling factor F(o)	11	4	0.07	55.05	+	1.48E-03
↳proton-transporting ATP synthase complex, coupling factor F(o)	13	4	0.09	46.58	+	2.87E-03
↳proton-transporting two-sector ATPase complex, proton-transporting domain	23	4	0.15	26.33	+	2.67E-02
↳proton-transporting two-sector ATPase complex	53	6	0.35	17.14	+	2.43E-03
↳membrane protein complex	1215	29	8.03	3.61	+	2.17E-06
↳protein-containing complex	5383	85	35.56	2.39	+	1.48E-15
↳proton-transporting ATP synthase complex	24	6	0.16	37.85	+	2.45E-05
↳intracellular part	14422	130	95.27	1.36	+	9.62E-10
↳intracellular	14699	130	97.1	1.34	+	7.85E-09
↳cell part	17007	135	112.35	1.2	+	1.06E-05
↳cell	17034	136	112.52	1.21	+	1.55E-06
↳mitochondrial proton-transporting ATP synthase complex	23	6	0.15	39.49	+	1.91E-05
↳inner mitochondrial membrane protein complex	147	21	0.97	21.63	+	1.38E-18
↳mitochondrial inner membrane	443	24	2.93	8.2	+	4.13E-12
↳mitochondrial membrane	674	27	4.45	6.06	+	8.48E-11
↳organelle membrane	2935	41	19.39	2.11	+	2.33E-03
↳organelle part	9480	110	62.62	1.76	+	2.86E-13
↳organelle	13455	129	88.88	1.45	+	2.86E-12
↳membrane-bounded organelle	12364	123	81.67	1.51	+	1.80E-11
↳mitochondrial envelope	713	27	4.71	5.73	+	3.15E-10
↳mitochondrial part	1008	29	6.66	4.36	+	2.88E-08
↳mitochondrion	1568	32	10.36	3.09	+	1.17E-05
↳cytoplasmic part	9646	111	63.72	1.74	+	2.80E-13
↳cytoplasm	11495	122	75.93	1.61	+	6.25E-14
↳intracellular membrane-bounded organelle	10846	108	71.65	1.51	+	2.57E-07
↳intracellular organelle	12625	124	83.4	1.49	+	2.78E-11
↳intracellular organelle part	9183	108	60.66	1.78	+	3.90E-13
↳organelle envelope	1161	29	7.67	3.78	+	7.73E-07
↳envelope	1161	29	7.67	3.78	+	7.73E-07
↳organelle inner membrane	503	24	3.32	7.22	+	6.32E-11
↳mitochondrial membrane part	235	22	1.55	14.17	+	9.86E-16
↳mitochondrial protein complex	279	21	1.84	11.39	+	4.73E-13
complex of collagen trimers	20	5	0.13	37.85	+	3.96E-04
↳supramolecular complex	966	20	6.38	3.13	+	8.81E-03
↳extracellular region part	3491	52	23.06	2.25	+	4.46E-06
↳extracellular region	4302	55	28.42	1.94	+	2.84E-04
↳extracellular matrix component	50	5	0.33	15.14	+	3.30E-02
mitochondrial respiratory chain complex Ⅳ	16	4	0.11	37.85	+	6.48E-03
↳mitochondrial respiratory chain	95	14	0.63	22.31	+	7.14E-12
↳respiratory chain	105	14	0.69	20.18	+	2.74E-11
↳respiratory chain complex Ⅳ	21	4	0.14	28.83	+	1.87E-02
↳cytochrome complex	36	6	0.24	25.23	+	2.62E-04
↳respiratory chain complex	90	12	0.59	20.18	+	2.14E-09
cytosolic small ribosomal subunit	49	10	0.32	30.89	+	2.68E-09
↳small ribosomal subunit	76	10	0.5	19.92	+	1.86E-07
↳ribosomal subunit	199	13	1.31	9.89	+	1.47E-06
↳ribonucleoprotein complex	871	19	5.75	3.3	+	7.39E-03
↳ribosome	252	13	1.66	7.81	+	2.35E-05
↳intracellular non-membrane-bounded organelle	4197	51	27.72	1.84	+	4.96E-03
↳non-membrane-bounded organelle	4204	51	27.77	1.84	+	5.22E-03
↳cytosolic ribosome	123	13	0.81	16	+	4.31E-09
↳cytosolic part	259	13	1.71	7.6	+	3.23E-05
↳cytosol	5077	64	33.54	1.91	+	2.07E-05
mitochondrial respiratory chain complex Ⅰ	52	6	0.34	17.47	+	2.18E-03
↳respiratory chain complex Ⅰ	52	6	0.34	17.47	+	2.18E-03
↳NADH dehydrogenase complex	52	6	0.34	17.47	+	2.18E-03
↳oxidoreductase complex	113	9	0.75	12.06	+	1.15E-04
polysome	76	6	0.5	11.95	+	1.87E-02
collagen trimer	92	7	0.61	11.52	+	4.45E-03
focal adhesion	404	15	2.67	5.62	+	1.30E-04
↳cell-substrate adherens junction	407	15	2.69	5.58	+	1.43E-04
↳adherens junction	538	16	3.55	4.5	+	9.06E-04
↳anchoring junction	554	16	3.66	4.37	+	1.33E-03
↳cell-substrate junction	411	15	2.71	5.52	+	1.62E-04
actin cytoskeleton	490	13	3.24	4.02	+	3.54E-02
supramolecular polymer	965	20	6.37	3.14	+	8.67E-03

extracellular exosome	2095	41	13.84	2.96	+	1.66E-07
↳extracellular vesicle	2115	42	13.97	3.01	+	5.72E-08
↳vesicle	3783	46	24.99	1.84	+	1.92E-02
↳extracellular organelle	2117	42	13.98	3	+	5.89E-08
↳extracellular space	3304	52	21.83	2.38	+	6.23E-07
intracellular organelle lumen	5222	61	34.5	1.77	+	1.04E-03
↳organelle lumen	5222	61	34.5	1.77	+	1.04E-03
↳membrane-enclosed lumen	5222	61	34.5	1.77	+	1.04E-03
Unclassified	2312	1	15.27	0.07	-	0.00E+00
