# Supplementary material for: Downregulation of mitochondrial biogenesis by virus infection triggers antiviral responses by cyclic GMP-AMP synthase
Source: PLoS Pathog. 2021 Oct 14;17(10):e1009841. doi: 10.1371/journal.ppat.1009841 (PMC8516216; doi:10.1371/journal.ppat.1009841)
Supplement: Attachment — Submitted filename: Responses to Reviewers comments.docx [file ppat.1009841.s012.docx]

**Reviewer #1**

We are grateful to Reviewer #1 for their critical comments and useful suggestions that have helped us to improve our paper. As indicated in the responses that follow, we have taken all these comments and suggestions into account in the revised version of our paper.

**Part I - Summary**

Reviewer #1: In their study entitled “Downregulation of mitochondrial biogenesis by virus infection triggers antiviral responses by cyclic GMP-AMP synthase” Sato et al. provide data that infection with measles virus (MeV), an RNA virus, causes mitochondrial elongation associated with release of mtDNA into the cytosol. Furthermore, they show that in addition to typical RNA innate immune activation, that the mtDNA release is accompanied by activation of the cytosolic DNA sensor cGAS, both in vitro and in vivo. Host cells infected with MeV show a downregulation of mitochondrial respiration and matrix genes, which the authors interpret as reduced mitochondrial biogenesis, and then show that knock-down of PGC-1a is sufficient to cause mtDNA release and cGAS-IFN signaling in MCF7 cells. They then determine that some additional, but not all, RNA and DNA viruses lead to downregulation of mitochondrial genes and induction of mtDNA-mediated activation of cGAS-STING pathway. This is a very interesting and important study. If true, this is the first implication of mitochondrial biogenesis per se in mtDNA-mediated innate immune signaling and implicating mtDNA as an agonist of host anti-viral signaling for RNA viruses more broadly is important. However, additional data and experiments are needed to solidify the authors’ main conclusions.

**Part II – Major Issues: Key Experiments Required for Acceptance**

Reviewer #1:

1) The authors main conclusion, which is even reflected in the title, is that downregulation of “mitochondrial biogenesis” is the key feature that drives mtDNA release and subsequent cGAS activation during MeV infection. However, the authors never actually never measure or perturb mitochondrial biogenesis in the infected cells. They instead observe that the mitochondria are hyperfused and show that reducing fusion by depletion of MFN1 reduced mtDNA-mediated cGAS signaling. This experiment shows that “mitochondrial dynamics” is involved, not “mitochondrial biogenesis.” Thus, the conclusions about biogenesis are based mainly, if not exclusively, on the gene signatures observed. The authors need to better characterize the mitochondrial phenotype in the infected cells, with a goal of demonstrating an effect on biogenesis. For example, is mitochondrial abundance lower in the infected cells, as predicted if biogenesis is inhibited. This can be measured with comparative westerns of mitochondrial and other housekeeping genes, by flow cytometry with mitochondrial potential-independent dyes, etc. Given the gene signature includes respiratory genes, the authors should also address mitochondrial membrane potential and OXPHOS activity, which should also be downregulated if biogenesis is reduced. Finally, is PGC-1a, TFAM and other or other biogenesis genes downregulated in the infected cells at the RNA and protein level? Finally, can overexpression of PGC-1a in the infected cells prevent mtDNA-mediated signaling as predicted by their model?

Answer

Following to Reviewer #1’s suggestions, we performed additional experiment to measure mitochondrial abundance by flow cytometry with MitoGreen, which is a mitochondrial mass-dependent dye, and successfully detected lower abundance of mitochondria post MeV infection (S1B Fig). In addition, we measured mitochondrial membrane potential using membrane potential dependent dye (JC-10), and decrease of the potential after MeV infection was detected (S1C Fig). Related to Reviewer #1’s comment 3), we further confirmed that the total amount of mtDNA in cells was decreased by MeV infection (S1D Fig). Taken together, we concluded that the downregulation of mitochondrial biogenesis by MeV infection is substantial.

We measured expression level of PGC-1a and TFAM in MeV-infected cells by RT-qPCR, and confirmed no alteration of their expression (S3C Fig.), which are predicted by our previous microarray analysis data [Ref. 13, 15]. Thus, we consider that the downregulation of mitochondrial biogenesis by MeV infection is not triggered by decrease of PGC-1a or TFAM directly.

In addition, we performed overexpression of PGC-1a during MeV infection to confirm whether it prevents mtDNA-release. However, unexpectedly the cells in which PGC-1a is overexpressed by expression plasmid apparently showed wrong condition by MeV infection, thus unfortunately we could not evaluate intracellular event. In Fig. 3, we utilized knockdown of PGC-1a as a model of downregulation of mitochondrial biogenesis, and independent of MeV infection as described above, thus overexpression of PGC-1a might not compensate for the mitochondrial biogenesis after MeV infection.

2) Related to point #1 above, to implicate mitochondrial biogenesis as sufficient for mtDNA-mediated signaling, the authors do knock-down PGC-1a and show that this instigates mtDNA release and cGAS signaling. To my knowledge this is the first time that reducing this pathway has been shown to be sufficient to mediate mtDNA innate immune signaling. Accordingly, this needs to be iron clad. First, similar direct measures of mitochondrial biogenesis and activity (not just qPCR of genes) needs to be demonstrated in this experiment (as described above) to show that the KD of PGC-1a leads to lower mitochondrial mass as predicted. Also, does this lead to mitochondrial hyperfusion as seen in the MeV-infected cells, which would corroborate in the KD and infection models? In addition, why was this experiment done in MCF7 cells? To make a stronger conclusion that reduced mitochondrial biogenesis is sufficient for mtDNA release and innate immune sensing, other cell types, including non-transformed cells or immune cells, should be tested. Finally, does KD of PGC-1a alone provide an anti-viral effect as predicted?

Answer

Following to the comment, we confirmed the lowering of mitochondrial membrane potential after MeV infection in PGC-1a KD cells in a same manner as above (S3Fig D).

On the other hand, apparent hyperfusion of mitochondria by PGC-1a KD was not observed. As shown in Fig. 3D, Mfn1 KD cells also showed lower level of mtDNA release. It implies that downregulation of mitochondrial biogenesis itself may cause mtDNA release at low level without hyperfusion. Further studies will be required to clarify the detail of hyperfusion and mtDNA release.

Wild type MeVs, including MeV-HL strain we utilized in this paper, use two receptors; SLAM on immune cells and nectin-4 on polarized epithelial cells. In the present study, we aimed to elucidate the consequence of downregulation of genes expression related to mitochondria in an epithelial cell line (293SLAM cells) (Fig. 1A). On the other hand, as described in Discussion, a lymphoid cell line (COBL cells established from human T lymphoid cells), which express SLAM, showed little alteration of gene expression after MeV infection in microarray analysis in our previous paper [Ref. 13, 15]. Thus, we did not perform a series of experiments with immune cells. Importantly, nectin-4 is little expressed on normal tissues in the body, while frequently upregulated on various cancer cells. Therefore, we utilized nectin-4 positive cancer cell lines or non-immune cell lines stably expressing SLAM which were generated artificially (293SLAM cells and Vero-hSLAM cells) in this study. Among them, we used MCF7 cells mainly, because MCF7 cells infected with MeV form little syncytia, thus preferable for many experiments in this paper, such as detection of cytoplasmic mtDNA and flow cytometry. Other cells form multinuclear giant cells by MeV infection, thus we used them for observation of hyperfused mitochondria mainly.

In S3C Fig., we have shown upregulation of ISGs as antiviral effect by PGC-1a KD. We further confirmed that the growth of MeV in PGC-1a KD cells is lower than normal cells. However, we could not determine whether the lower growth of MeV is a consequent of upregulation of ISGs or unknown effect of PGC-1a KD (because overexpression of PGC-1a affect cellular condition after MeV, as described above), thus the data is not described.

3) The authors use cellular fractionation and cGAS immunoprecipitation to demonstrate the presence of mtDNA in the cytosol. First, it is critical that the authors show the western blots demonstrating the purity of the fractions used for this experiment in all cases (i.e. during infection and PGC-1a KD, etc.). Second, others have shown that mtDNA release involves enlarged or clustered mtDNA nucleoids and mtDNA depletion. The authors should visualize mtDNA with picogreen or an anti-DNA antibody to address nucleoid morphology and perhaps also see it outside of mitochondria as predicted, and assess mtDNA copy number, which should also be depleted if mitochondrial biogenesis is downregulated.

Answer

Following to the reviewer’s comment, we added the digitonin fractionation by western bloting in S2A Fig. We also performed similar western blotting in MeV infected cells (Fig. 2A), Mfn1 KD cells (Fig. 3D), ActD/UV/HA-Mfn1 cells (Fig. 3G) and PGC-1a KD cells (Fig. 3I), but all results showed no difference, thus we omit them.

We tried to observe alteration of nucleoid morphology and mtDNA outside of mitochondria in MeV-infected cells with picogreen, but could not. In the present study, the amount of cytoplasmic mtDNA released by MeV infection was significantly low compared to the total amount of mtDNA in the cells, 0.08-0.14 %, at 3 dpi. We think that the small amount of mtDNA release is not sufficient for inducing morphological change of nucleoid nor microscopic observation of cytoplasmic mtDNA, but enough for activation of cGAS.

As described above, we measured total amount of mtDNA in cells after MeV infection, and showed in S1D Fig.

4) The authors propose “a two-step induction of antiviral responses; at an early phase of infection, viral RNA replication is detected rapidly by an RNA sensor, while during the late phase of infection, mitochondrial downregulation accompanied by mtDNA liberation causes prolonged IFN-β and ISG production. These findings uncover a novel host strategy of the defense system for suppressing viral propagation.” This is a really important concept, thus, can the authors perform a time course of MeV infection, in which they can show that activation of the RNA sensor pathways precedes that of the mtDNA sensing pathway?

Answer

Following to your suggestion, we performed additional experiment using Luciferase assay driven by interferon signaling response element (ISRE), which is activated by IRF3 to detect antiviral responses, and described in Fig. 2D. As predicted, MeV infection induced rapid ISRE activation (from 6 hpi) which might be caused by RNA sensor. In addition, we successfully detected phosphorylation of STING, which is a hallmark of cGAS activation, after diminishing of ISRE activation from 1 dpi. These results will support our concept that the cGAS activation by liberation of mtDNA is a host strategy against sustain of MeV replication which causes damage of maintain of cells by comprehensive downregulation of housekeeping genes including mitochondrial biogenesis.

**Part III – Minor Issues: Editorial and Data Presentation Modifications**

Reviewer #1:

• In the discussion the authors use a term called “mitochondrial thickness”. This term is undefined and not very clear from the images presented (e.g. Fig 1B).

Answer

We tried to quantify the thickness of mitochondria in addition to the length, but could not well. We deleted the sentence including “mitochondrial thickness” from the Discussion.

• In Figure 3D (cytosolic mtDNA measurement) is the difference between mock and MeV under control or Mfn 1 downregulation significant? Please indicate statistical analysis used.

Answer

Following to your comment, we added statistical analysis between mock and MeV (Fig. 3D).

• Figure 4A: X and Y axes are no labelled.

Answer

We labelled X axis (days post inoculation; dpi) and Y axis (survival rate).

• In Figure 5B, SeV and MVA infection seems to be dramatically lower than that of RSV, probably explaining the lack of mitochondrial elongation? Is this confounding?

Answer

In Fig. 5B, we selected Figures of SeV and MVA, in which all the cells were infected same as RSV, because mock-treated Hep-2 cells were shown independently.

On the other hand, we omitted “mock” of HeLa and MDCK cells to reduce the number of panels in Fig. 5B, and selected figures which contain both noninfected and infected cells. We exchanged the Figure of VSV and CPV for a new one which are lower magnification and contain more cells. In Fig. 5C, we analyzed again with ~ 30 infected cells per each virus with triplicate, as with other conditions, and the elongation of mitochondria by RSV and MVA, but not by SeV, VSV and CPV are evaluated statistically.

• Scale bars should be indicated in Figures 2D, 3E, 5B, S1B & C.

Answer

We added scale bar.

**Reviewer #2**

We are grateful to Reviewer #2 for their critical comments and useful suggestions that have helped us to improve our paper. As indicated in the responses that follow, we have taken all these comments and suggestions into account in the revised version of our paper.

**Part I - Summary**

Reviewer #2: Sato et al. describe in this manuscript the involvement of the cytoplasmic DNA sensor cGAS in interferon induction and setup of an antiviral state in response to an infection with an RNA virus, measles virus (MeV). Upon infection they observe morphological changes in mitochondria leading to the release of mitochondrial DNA into the cytoplasm. These DNA molecules then activate cGAS and lead to increased IFN production. The authors provide mechanistic insights into the molecular processes by employing siRNA knock-down and overexpression strategies for cGAS and genes involved in mitochondria function, Mfn1 and PGC-1alpha. They then show that cGAS contributes to establishment of an antiviral response against MeV in an animal model. Finally, the authors extend their observations to several other RNA and DNA viruses. Importantly, while the pneumovirus respiratory syncytial virus (RSV) showed cGAS-dependent IFN induction, the murine paramyxovirus Sendai virus (SeV) and the rhabdovirus vesicular stomatitis virus (VSV) showed no effects of cGAS. Similarly, modified vaccinia virus Ankara (MVA), a dsDNA poxvirus, activated cGAS through this mitochondrial pathway, whereas a ssDNA virus, canine parvovirus (CPV) did not.
The contribution of cGAS to antiviral responses against negative-strand RNA viruses (NSV) is novel. The authors suggest that this contribution is a specific cellular response to certain NSV. Although a common feature of viruses involving this pathway is downregulation of gene expression of genes involved in mitochondria biogenesis, the panel of viruses tested leaves a big question mark on why some viruses exhibit this phenotype (MeV, RSV, MVA), while others do not (VSV, SeV, CPV).
From the immunofluorescence stainings provided here it becomes apparent that MeV infection induces mitochondrial hyperfusion leading to release of mitochondrial DNA into the cytoplasm. The authors suggest that this event is a specific antiviral response. However, it may as well be a manifestation of virus-induced cell death. The authors use a wild type MeV which has a highly fusogenic membrane fusion apparatus consisting of H and F proteins. The virus induces the formation of very large syncytia, which are known to change morphology and rapidly lose integrity. Mitochondrial hyperfusion and release of mtDNA may be an indirect consequence of the overall loss of cell integrity. The use of Actinomycin D and UV irradiation, which induce mitochondrial stress but not cytosolic mtDNA release, support this hypothesis.
While the authors use specific siRNA knock-down approaches to show synergy between the RIG-I-like receptor/MAVS-mediated and the cGAS-mediated IFN induction in response to MeV infection, an apparent question arises whether MAVS-mediated signaling processes are involved in changing mitochondria morphology. Since MAVS is incorporated into the outer mitochondrial membrane, assembly of the RLR signaling complex at these sites may play a role in mitochondria hyperfusion and mtDNA release. Unfortunately, while possibly important in the context of this study, this is addressed neither in experiments nor in the discussion of this manuscript.
The mouse experiments finally show some contribution of cGAS to MeV pathogenesis. The authors choose a model of intracranial infection of MeV into wild type, MAVS-/- and cGAS-/- mice. Surprisingly, while the authors claim to have a lethal infection model (line 204), 60% of wild type mice survive this infection. Notably, in the cited publication, MeV infection using the same virus and mouse strains caused 100% lethality (Arai et al. J Gen Virol. 2017;98(7):1620-9). Or was it intentional to use sublethal infections in wild type mice?

Answer

As Reviewer #2 mentioned, it is difficult to conclude why MeV, RSV and MVA trigger mtDNA-cGAS pathway similarly, because these viruses possess no common viral component nor characteristic. We consider that the downregulation of mitochondrial biogenesis is one of the intrinsic features of respective viruses, and these viruses cause the downregulation by individual purpose for exhibition of the specific pathogenicity. On the other hand, the host cells catch these mitochondrial stresses as a same signal, and fulfill host response by liberation of mtDNA to cytoplasm as an adaptive response.

As described in Discussion, we are currently interested in the host transcriptional regulatory network after MeV infection, which includes trigger of mitochondrial downregulation, and attempting to elucidate the whole aspect using novel analysis method. This study may reveal unknown mechanism for the mitochondrial downregulation by these viruses from the standpoint of host gene expression network.

We answered to other comments in Part I Summary section as answers to Part II Major issue, described below.

**Part II – Major Issues: Key Experiments Required for Acceptance**

Reviewer #2:

1. Influence of MAVS pathway on mitochondrial hyperfusion and cGAS activation: Is mitochondrial hyperfusion after MeV infection dependent on RLR-signaling? This could easily be addressed by measuring mitochondrial hyperfusion in MAVS knock-down cells vs. wild type cells.

Answer

Following to your suggestion, we performed additional experiment to observe mitochondrial morphology after MeV infection in MAVS-knockdown (KD) cells. We confirmed that MAVS-KD cells also formed hyperfused mitochondrial morphology same as wild-type cells (S2C Fig), suggesting that the depletion of MAVS pathway did not influence on the mitochondrial morphological change, and the formation of hyperfused mitochondria is different process from assembly of RLR signaling complex on the outer mitochondrial membrane.

2. Dependence on syncytia formation: The authors should provide a more detailed kinetic of mitochondria hyperfusion and correlate their findings with the kinetics of syncytia formation. Also, comparison of wild type and vaccine MeV strains, which have different fusogenic potentials, will offer valuable insights. Alternatively, treatment of cells with fusion imhibitory peptide after MeV infection would address this question.

Answer

Based on your comment, we observed mitochondrial morphology in multinuclear giant cells which were formed by coexpression of MeV-F and H proteins. As a result, elongation of mitochondria was not observed (S3B Fig). On the other hand, MeV-infected MCF7 cells, which form little large syncytia, showed hyperfusion of mitochondria as shown in S1B Fig. Therefore syncytia formation and mitochondrial hyperfusion might be distinct event. In the present study, we have not focused on the syncytia formation for hyperfusion of mitochondria, thus we omit the experiment for kinetics of syncytia formation.

3. Mouse experiments: Although introduced as a lethal infection model (line 204), 60% of wild type mice survive infection by day 13. Why was the experiment terminated at this time? Would the data and their interpretation have changed if mice were observed for a longer period, i.e. would more mice have succumbed to infection after day 13? Also, according to materials and methods, the authors injected a total volume of 30 ul intracranially into mice with at least 7 grams body weight. This seems a very high amount. Could this volume be considered to induce traumatic brain injury by itself? How was it injected: Free-hand or using a stereotactic device and microfluid pump to control injection flow rate?

Answer

As the reviewer indicated, the CAM-R strain of MeV we utilized shows 100% lethality when 1.4 x 10^4 TCID50 of virus was inoculated. While our aim in this study is to reveal contribution of cGAS, in addition to MAVS, as an antiviral factor on the MeV infection. Thus, we needed to show upregulation of lethality by MeV in cGAS or MAVS knockout mice, and used sublethal dose of MeV in wild-type mice intentionally. We decided sublethal dose of MeV in wild-type mice by a preliminary test, and used it (1.0 x 10^4 TCID50). In addition, we think that 13 dpi is appropriate to judge the implication of these antiviral factors.

In our laboratory, we routinely inject 25-30 ul of virus solution to neonatal mice intracerebrally using craniopuncture under mild anesthesia without brain injury. (Ref 41, Takenaka et al., J. Virol 90(20) 9285-9292, 2016). The injection was performed by free hand at the same site, at the vertex of a regular triangle joining the right eye and ipsilateral ear, beside the cranial suture.

**Part III – Minor Issues: Editorial and Data Presentation Modifications**

Reviewer #2:

Fig. 2B: What are the absolute levels of mtDNA enrichment in contrast to the presented relative enrichment values?

Answer

The absolute levels of mtDNA in Fig. 2B have been undetermined. In this study, the amounts of cytoplasmic mtDNA induced by MeV infections were significantly low compared to the total amounts of mtDNA in cells, 0.08-0.14 %, at 3 dpi. Thus, we think that similar level of cytoplasmic mtDNA was presented in Fig. 2B.

Fig. 2C: After MeV infection, are the IFNbeta mRNA levels in MAVS-cGAS double-knockdown cells significantly lower than MAVS knockdown alone? The indicated asterisks seem to compare each sample to NC control.

Answer

We calculated the p value of MAVS knockdown cells vs MAVS-cGAS double-knockdown cells after MeV infection, and the result was “ns”. We deleted the description “double knockdown synergistically abrogated IFN-b production”.

Figs. 2 and 5: Use of ddC: What are the effects of blocking mtDNA replication on cell survival and virus replication? If mitochondria are not functioning properly this may affect cell viability and virus replication in general.

Answer

Following to your comment, we confirmed the cell viability and virus titer after ddC treatment. As a result, ddC treatment and resultant depletion of mtDNA did not affect on them (S2 D Fig). These results indicated that the decrease of IFN-b expression after MeV infection by ddC treatment (Fig. 2D) is not consequence of decreased cell viability and virus growth.

Fig. 5B: No images of uninfected HeLa and MDCK cells are provided to assess mitochondria structures.
S3 Figure: The labels of the subpanels do not align with what is referred to in the text (Is S3B supposed to be the diagram next to the IF images? Is S3C the bar diagram on the bottom or all three bar diagrams labelled as S3B?) Please correct.
lines 171-175: This sentence is confusion. Please edit.

Answer

To reduce the number of panels in Fig. 5B, we omitted “mock” of HeLa and MDCK cells and selected figures which contain both noninfected and infected cells. We exchanged the Figure of VSV and CPV for a new one which are lower magnification and contain more cells. In Fig. 5C, we analyzed again with ~ 30 infected cells per each virus with triplicate, as with other conditions, and the elongation of mitochondria by RSV and MVA, but not by SeV, VSV and CPV are evaluated statistically.

We corrected the labels of subpanels in S3Fig.

We edited the sentence corresponding to original line 171-175.
