# Supplementary material for: Downregulation of mitochondrial biogenesis by virus infection triggers antiviral responses by cyclic GMP-AMP synthase
Source: PLoS Pathog. 2021 Oct 14;17(10):e1009841. doi: 10.1371/journal.ppat.1009841 (PMC8516216; doi:10.1371/journal.ppat.1009841)
Supplement: Attachment — Submitted filename: Responses to Reviewers 2nd.docx [file ppat.1009841.s013.docx]

**Reviewer #1**

We are grateful to Reviewer #1 for the useful comments. We answered all of these comments as described below, and added figures in the revised version of our paper. We hope that our responses are now sufficient to solve your questions.

**Part I - Summary**

Reviewer #1: The authors have provided substantial new data to bolster their conclusions and the manuscript is improved. However, there are some remaining issues related to the original critique that were not resolved and should be addressed:

**Part II – Major Issues: Key Experiments Required for Acceptance**

Reviewer #1: 1. In the PGC-1a knock-down cells only mitochondrial potential was measured. Mitochondrial membrane potential can change without any change in mitochondrial biogenesis/abundance. To better correlate this phenotype to the MeV phenotype, the same measures of mitochondrial abundance should be performed in both experiments.

Response

We performed FACS of PGC-1α knock-down cells with MitoGreen same as MeV-infected cells, but a decrease of mitochondrial mass was not significant.

Our previous studies (Ref 13-15) have suggested that MeV infection influences on various cellular transcriptional networks, which will affect on multiple signaling related to mitochondrial biogenesis, and as a consequent, many genes of mitochondrion are downregulated. Nevetheless, as described in S1B Fig, the decrease of mitochondrial mass under the condition was only ~30 %.

PGC-1α is a coactivator of transcription factor NRF-1 and 2, and considered to contribute to a restricted part of a huge mitochondrial biogenesis. Thus, the consequence of PGC-1α knockdown will not be reflected to the total mass of mitochondria. On the other hand, NRF-1 and 2 govern expression of proteins for mitochondrial oxidative phosphorylation which conjugate to an electron transport chain, thus the PGC-1α knockdown might cause the detectable decrease of mitochondrial membrane potential.

2. The authors have still not provided evidence that RNA sensing is occurring before mtDNA sensing as stated by their "two-step model." The ISRE reporter does does not distinguish between RNA and DNA innate immune sensing. Also, when the mitochondrial changes are occurring (early or late) is key to this model. Thus, temporal ordering of RNA signaling, mitochondrial changes and DNA sensing is still needed to bolster the authors conclusions with regard to the two-step model.

Response

To distinguish RNA sensing and DNA sensing, we detected phosphorylation of STING, and confirmed that the DNA sensing by cGAS-STING pathway occurs after early peak of ISRE (Fig. 2D). We think that the ISRE activation is certainly induced by regular RNA sensing.

Also we think it will be possible to discuss the temporal ordering of each event by combining each results in our studies.

For example, Mfn1 knockdown showed decrease of cytosolic mtDNA (Fig. 3D), indicating that the DNA sensing occurs subsequently to mitochondrial hyperfusion.

Furthermore, as described in the Introduction, our previous microarray analysis indicated that the rapid antiviral responses are induced at an early phase of MeV infection (6 hpi), which supports the peak of ISRE activation in Fig. 2D, while comprehensive downregulation of housekeeping genes including mitochondrion, which triggers hyperfusion of mitochondria, occurs at a late phase (24 hpi).

Therefore, it will be concluded that MeV infection activates RNA sensing at first, and then induces mitochondrial change by a consequence of downregulation of housekeeping genes, and subsequently induces DNA sensing via mtDNA-cGAS pathway.

3. The western blots showing purity of cytoplasmic fraction from all experiments should be shown even they all look the same.

Response

Following your comment, we added the figures as S2AFig, S3AFig and S3FFig.

**Part III – Minor Issues: Editorial and Data Presentation Modifications**
Please use this section for editorial suggestions as well as relatively minor modifications of existing data that would enhance clarity.

Reviewer #1: Figure S2a "nucleus" is misspelled

Response

We corrected the spell.

**Reviewer #2**

We are grateful to Reviewer #2 for the useful suggestion. As described below, we answered to your comment. We hope that the response is sufficient to resolve your question.

**Part I - Summary**

Reviewer #2: The revised manuscript by Sato et al. is greatly improved and addresses all of my previous concerns. The authors clearly show that measles virus, as well as RSV and MVA, induce the liberation of mitochondrial DNA at later stages during infection, which in turn causes activation of the cGAS/STING pathway. The in vivo experiments with cGAS-/- mice suggest that this pathway indeed contributes to the total antiviral response initiated by the host. The manuscript is well written and the data support the authors' conclusions.
It is still unclear why and how this downregulation of mitochondrial biogenesis and release of mitochonrial DNA occurs during MeV infection, but this will clearly require extensive research beyond the scope of this manuscript.

**Part II – Major Issues: Key Experiments Required for Acceptance**

Reviewer #2: none

**Part III – Minor Issues: Editorial and Data Presentation Modifications**

Reviewer #2: I have only one issue the authors should consider:
The qPCR data in Fig. 4C and S4 is presented as "Gene XYZ / MeV N". However, as shown in Fig. 4B, MeV N greatly differs between wild type and cGAS-/- mice (at least some of them). Therefore, the calculated lower values for the Gene XYZ / MeV N ratios in cGAS-/- mice may mostly be due to the increased MeV N expression. It would be more accurate to show all gene expression relative to the housekeeping gene (according to M&M 28S rRNA). In this case we could better assess whether there is less IFNb or other ISG expression observed in cGAS-/- mice.

Response

As Reviewer #2 pointed out, MeV-N mRNA greatly differ among individual mice, which indicates that MeV replication also varied.

In principal expression of IFN-β and ISGs are induced by MeV replication, thus the amount of IFN-β and ISGs productions are considered to correlate with MeV-N production.

Our purpose in these figures is that cGAS also contributes to IFN-β and ISGs expressions by MeV replication. Therefore, we needed to compare with the degree of IFN-β and ISGs expressions relative to MeV replication between wild-type and cGAS KO mice. If these expressions are described relative to the housekeeping genes, the expression level will not be linked to the MeV replication. Thus, we adopted this description.
